# Supplementary material for: Emittance preservation in a plasma-wakefield accelerator
Source: Nat Commun. 2024 Jul 19;15:6097. doi: 10.1038/s41467-024-50320-1 (PMC11271607; doi:10.1038/s41467-024-50320-1)
Supplement: Supplementary file 1 — Supplementary Information [file 41467_2024_50320_MOESM1_ESM.pdf]

Supplementary Information

Emittance preservation in a plasma-wakefield accelerator

C. A. Lindstrøm[1,2,*], J. Beinortaitė[1,3], J. Björklund Svensson[1], L. Boulton[1,4,5], J. Chappell[3], S. Diederichs[1,6], B. Foster[7], J. M. Garland[1], P. González Caminal[1,6], G. Loisch[1], F. Peña[1,6], S. Schröder[1], M. Thévenet[1], S. Wesch[1], M. Wing[1,3], J. C. Wood[1], R. D'Arcy[1] & J. Osterhoff[1]

[1] Deutsches Elektronen-Synchrotron DESY, Hamburg, Germany.

[2] Department of Physics, University of Oslo, Oslo, Norway.

[3] University College London, London, UK.

[4] Department of Physics, Scottish Universities Physics Alliance, University of Strathclyde, Glasgow, UK.

[5] The Cockcroft Institute, Daresbury, UK.

[6] John Adams Institute, Department of Physics, University of Oxford, Oxford, UK.

[7] Universität Hamburg, Hamburg, Germany.

*Corresponding author: c.a.lindstrom@fys.uio.no

Supplementary Figures

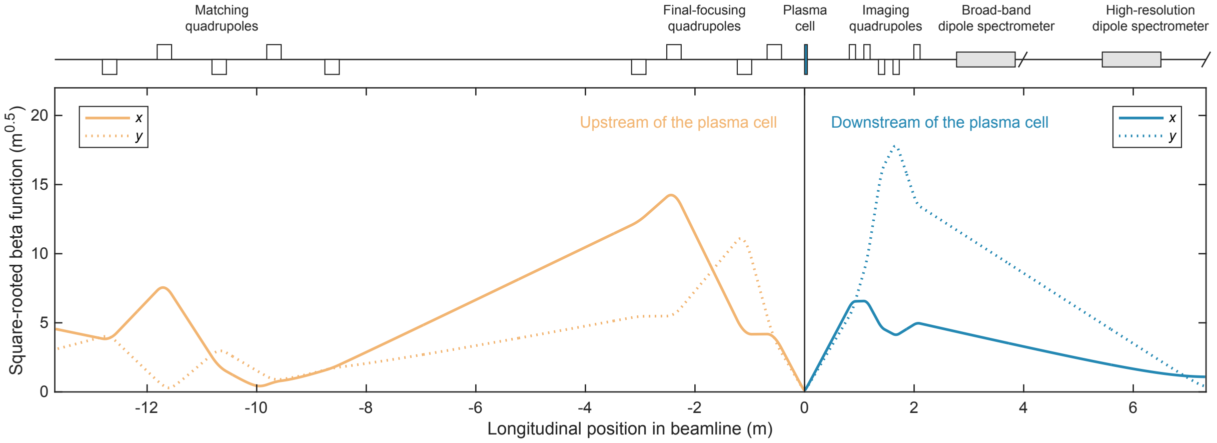

Supplementary Fig. 1 | Final-focusing and imaging optics. The electron bunches were strongly focused to match the beam size and beta function to the plasma-wakefield accelerator. Nine quadrupole magnets (white rectangles in the above beam-line sketch) were used for matching and final focusing upstream of the plasma cell (dark blue rectangle in the sketch). Here, the square root of the beta function in the horizontal (x; solid lines) and vertical (y; dotted lines) planes are shown; this quantity is proportional to the beam size. Downstream of the plasma cell, five quadrupoles were used for capturing the diverging electron bunches and point-to-point imaging them onto one of two spectrometer screens (black slanted lines in the sketch) after dispersion by a dipole magnet (grey rectangles in the sketch). Only the optics for the high-resolution-spectrometer screen, used for emittance measurements, is shown.

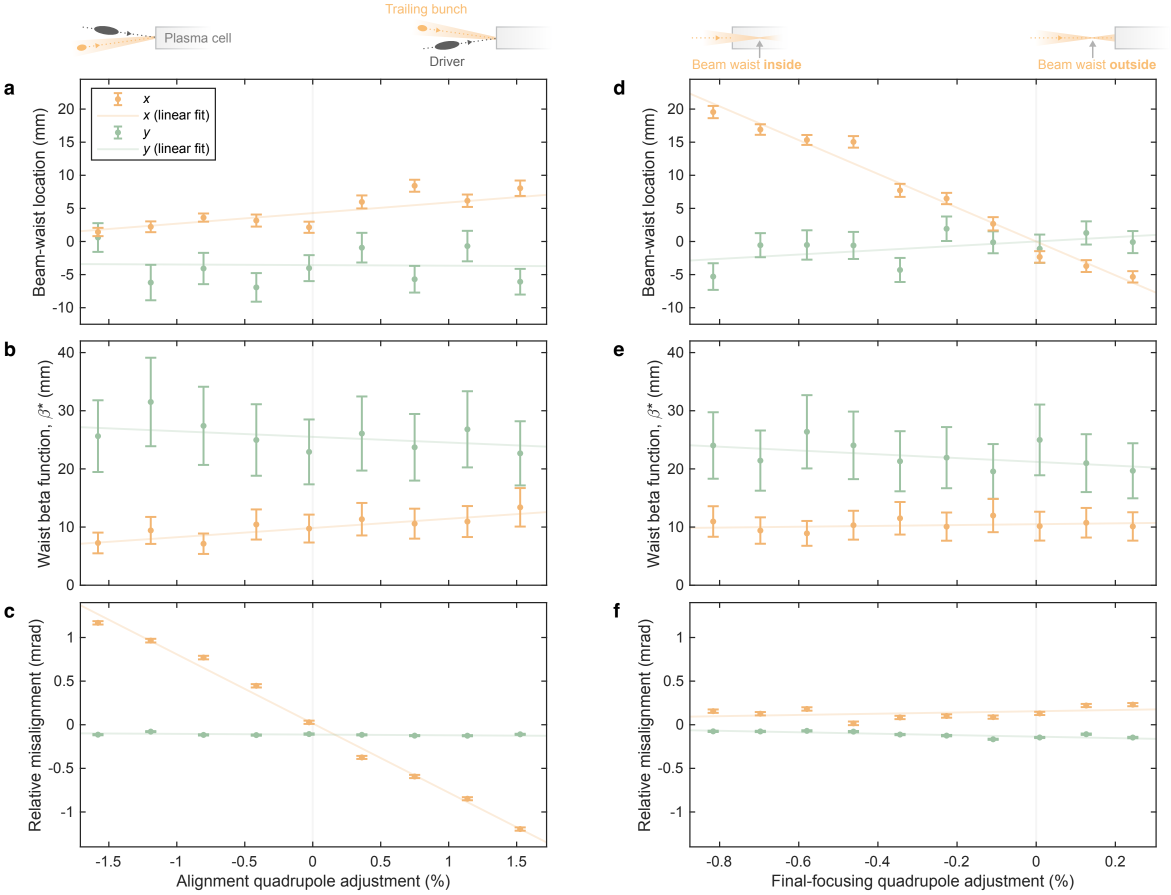

Supplementary Fig. 2 | Relative changes of the beam waist and alignment estimated using beam-position monitors. The distribution of beam trajectories jittering over multiple shots were used to measure relative changes in beam-waist location and beta function[36], as well as the misalignment between the driver and trailing bunches. **a**, The beam-waist location was measured for each step in the alignment scan used in Fig. 3, where the beam-waist location of the emittance-preserving operating point (Fig. 2) is defined as zero. A small relative shift in the horizontal plane (orange error bars) occurred when adjusting the strength of the alignment quadrupole (moves 1.6 mm downstream per 1%), while remaining approximately constant in the vertical plane (green error bars). In all plots, the points and error bars represent the best fit value and statistical uncertainty; 150 shots were used per data point. **b**, A similar change occurred for the horizontal-waist beta function (1.6 mm larger per 1%), while the vertical-waist beta function remained approximately constant. **c**, The relative alignment between the driver and trailing bunches, whose trajectories were measured separately, changed significantly in the horizontal plane (0.8 mrad per 1%), while remaining unchanged in the vertical plane. **d–f**, A similar measurement was performed for the scan of the beam-waist location shown in Fig. 4. Here, the horizontal-waist location changed significantly when changing the strength of a final-focusing quadrupole (moving 25 mm upstream per 1%), while the vertical-waist location moved negligibly (moving 3 mm downstream per 1%). The waist beta functions and relative misalignments also remained approximately constant.

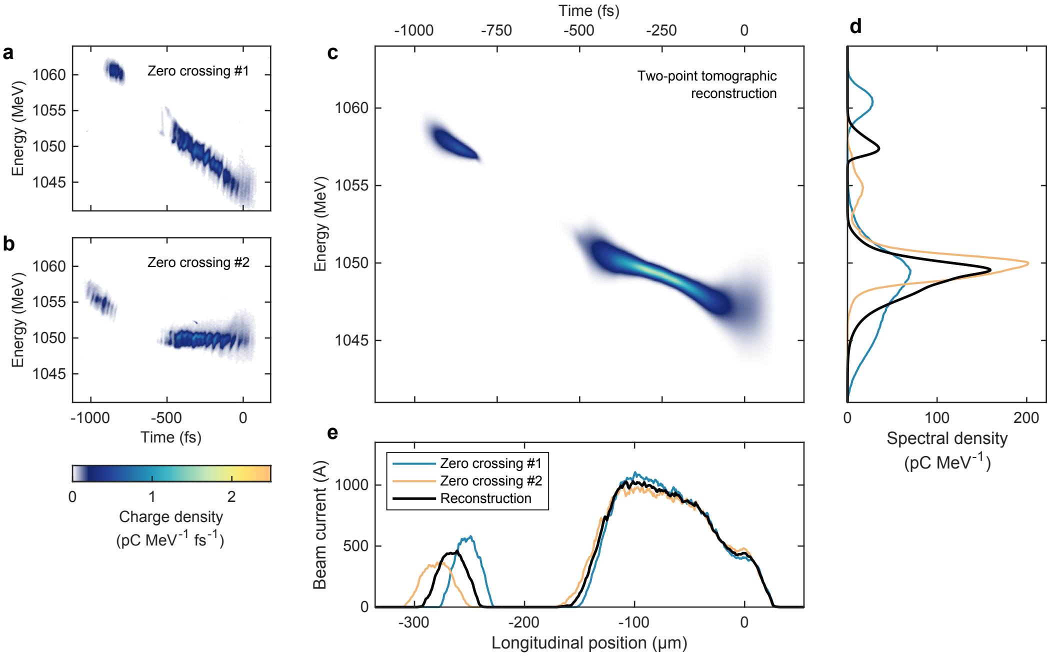

Supplementary Fig. 3 | Longitudinal-phase-space measurement. A transverse-deflection structure and a magnetic dipole were used to streak in time and disperse in energy, respectively, onto a scintillator screen. **a–b**, The measurement was performed in both zero crossings of the oscillating field. Microbunches can be observed in the streaked bunches. **c**, Using 50 shots from each zero crossing, a two-point tomographic reconstruction can be made; no microbunching is visible due to multi-shot averaging. Two bunches, a driver (bottom right) and a trailing bunch (top left), were created using a notch collimator in an energetically dispersive region. Here, the energy resolution of 0.64 MeV rms was removed by subtraction in quadrature. **d**, An energy projection shows that the two bunches are spectrally distinct. **e**, Similarly, the measured current profile indicates that the driver and trailing bunches are temporally distinct, with peak currents of 1.0 and 0.44 kA, respectively.

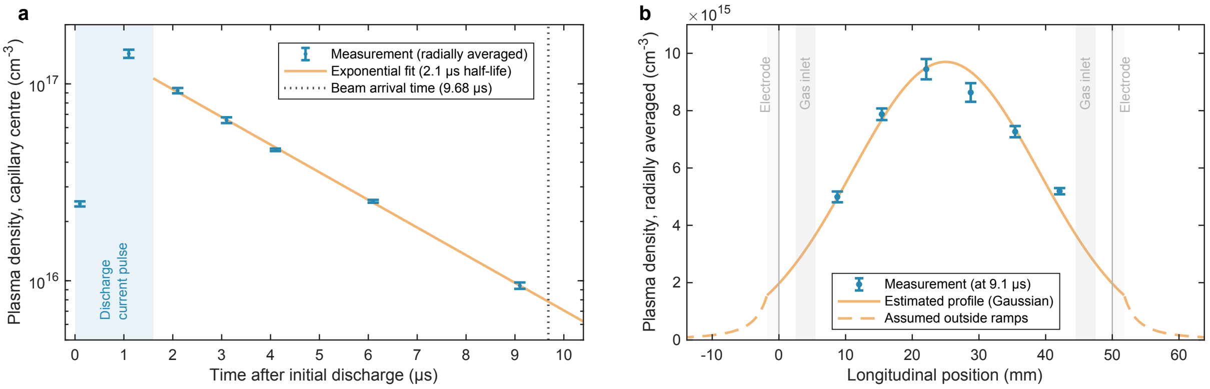

Supplementary Fig. 4 | Plasma-density evolution and longitudinal profile. a, The plasma density at the capillary centre (blue error bar) was measured based on H-alpha spectral-line broadening, where the error bars indicate the statistical fit error. This was integrated across a 7 mm-long region longitudinally and across the full radius of the capillary. After the discharge current pulse (blue area) stops, the central plasma density decays exponentially with a half-life of 2.1 μs (orange line). The beam arrived 9.68 μs after the initial discharge. **b**, Close to this time (at 9.1 μs) the measured longitudinal plasma-density profile (blue error bars) has a Gaussian-like shape inside the cell (orange curve). It was not possible to measure the low-density ramps outside the electrodes of the plasma cell—a polynomial shape was assumed in PIC simulations, emulating a cone-shaped expulsion. At the beam arrival time (0.58 μs later), the density profile is expected to retain its shape, but decay by a further 20% (to a peak of 8×10^{15} cm^{-3}). The on-axis density, as experienced by the beam, is expected to be approximately 50% higher than the measured radially averaged density (i.e., peaking at 1.2×10^{16} cm^{-3}).

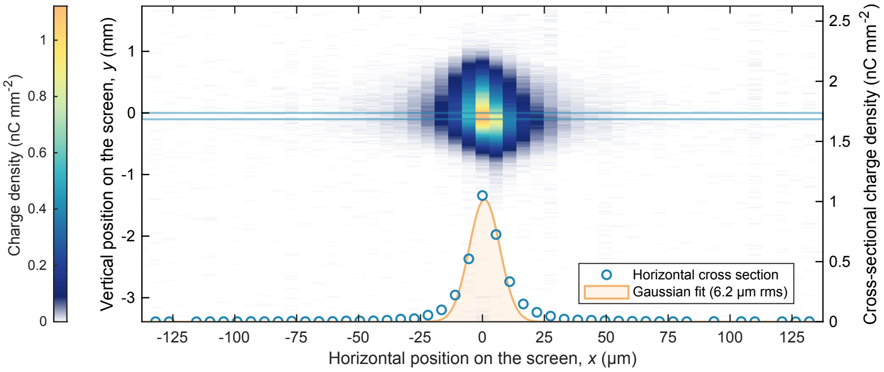

Supplementary Fig. 5 | Screen-resolution measurement for the high-resolution spectrometer. A tightly focused and highly collimated beam (with 20 pC of charge) was imaged onto the high-resolution spectrometer screen with a beam-imaging magnification of -1. Note that the image has unequal scales in the horizontal (bottom axis) and vertical planes (left axis). The central cross section in the horizontal plane (blue circles, right axis) shows that the point-spread function is Gaussian-like with extended tails. The pixel width corresponds to 5.5 μm on the screen. The fitted Gaussian distribution (orange area) has an rms of 6.2 μm, suggesting that the screen resolution is negligible compared to the beam sizes measured in the emittance measurements, which were operated with a magnification of -7.9.

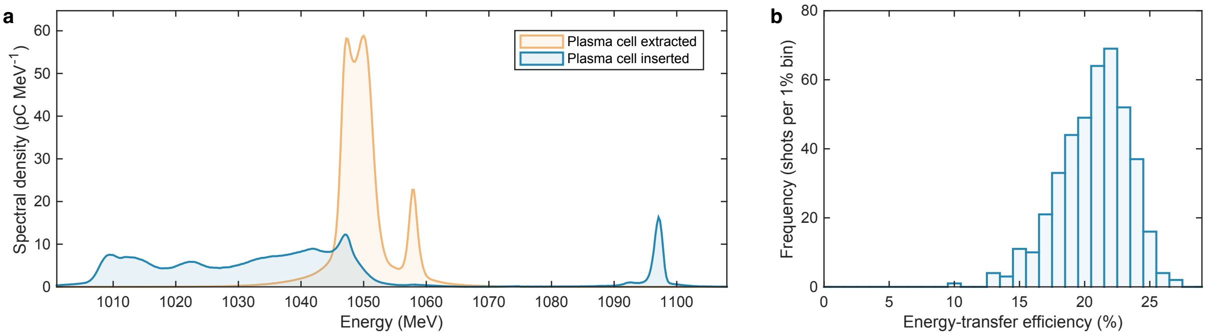

Supplementary Fig. 6 | Measurement of energy-transfer efficiency. a, Complete energy spectra were measured using the broad-band spectrometer, both while the plasma cell was extracted (orange area) and inserted (blue area). Here, the decelerated driver spectrum was reconstructed using an imaging-energy scan for increased accuracy. The accelerated trailing-bunch spectrum represents the shot with the highest peak spectral density. The energy spread appears higher (and spectral density lower) compared to Fig. 1f and Supplementary Fig. 3 due to a lower energy resolution, but this has a negligible effect on the calculated efficiency. **b**, Combining the energy lost by the driver (on average), as measured on the broad-band spectrometer, and the energy gained by the trailing bunch (shot-by-shot, not simultaneously), as measured on the high-resolution spectrometer (Fig. 2c–e), the energy-transfer efficiency of the emittance-preserving operating point can be calculated. A histogram shows the shot distribution of energy-transfer efficiency peaking at 22 ± 2.2%, and reaching a maximum of 27 ± 2.7%, where the quoted error arises from a systematic uncertainty of the driver energy loss, due to a 20% decrease in charge between the reconstructed cell-extracted and cell-inserted driver spectra.

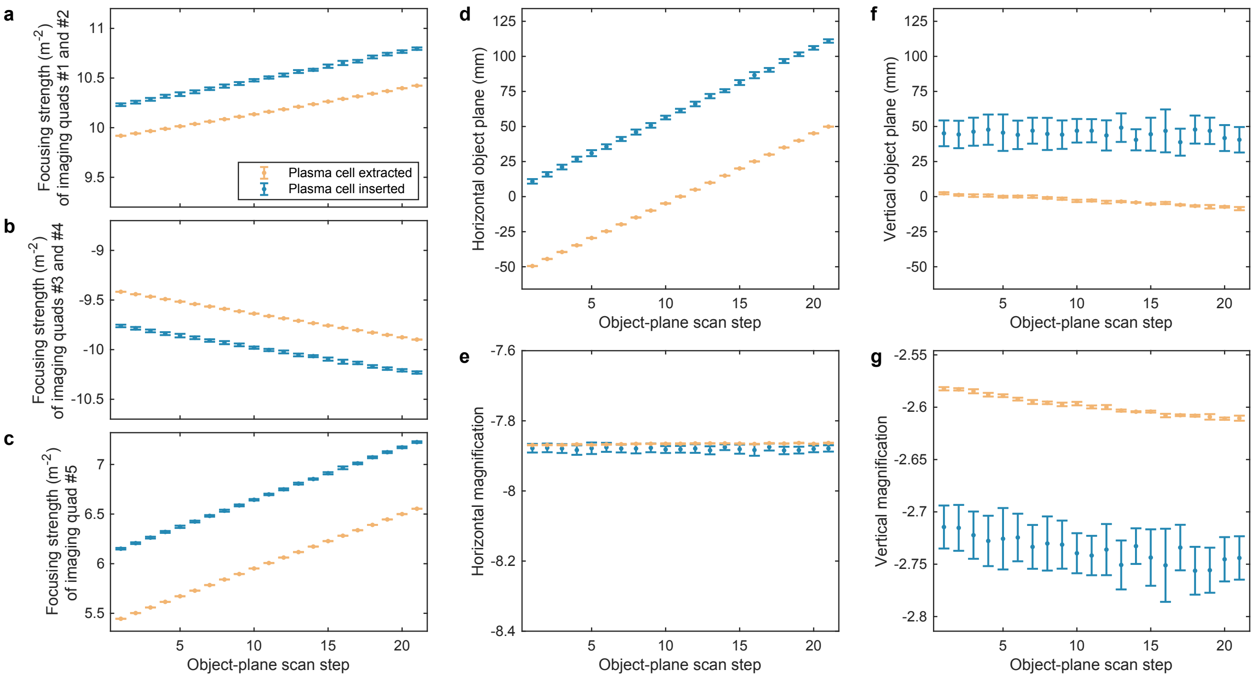

Supplementary Fig. 7 | Object-plane scan for emittance measurements. a–c, In order to scan the horizontal object plane while maintaining a constant horizontal magnification and constant vertical object plane, the current in each of the five imaging quadrupoles (three of which were independent) was calculated and precisely adjusted. The resulting focusing strengths, calculated for the mean trailing-bunch energy of each shot, are shown for the scans with plasma cell both extracted (orange error bars) and inserted (blue error bars), corresponding to Fig. 2b and 2c, respectively. In all plots, error bars indicate the mean and rms of the shots in each scan step. **d**, The object plane in the horizontal plane was scanned over a 100-mm range centred around the plasma entrance and exit. **e**, The horizontal magnification was kept constant at approximately -7.9. **f**, The vertical object plane was also kept constant at a location resulting in a small vertical beam size in the dispersive plane, to maintain high energy resolution. A small drift appears in the cell-extracted scan, but is sufficiently small to be negligible in the energy-spectrum measurement. **g**, Since only three quadrupole currents could be independently adjusted to satisfy the constraints on the horizontal and vertical object plane as well as the horizontal magnification, the vertical magnification could not be simultaneously controlled, but varied slightly around a value of -2.60 (plasma cell extracted) and -2.74 (plasma cell inserted).

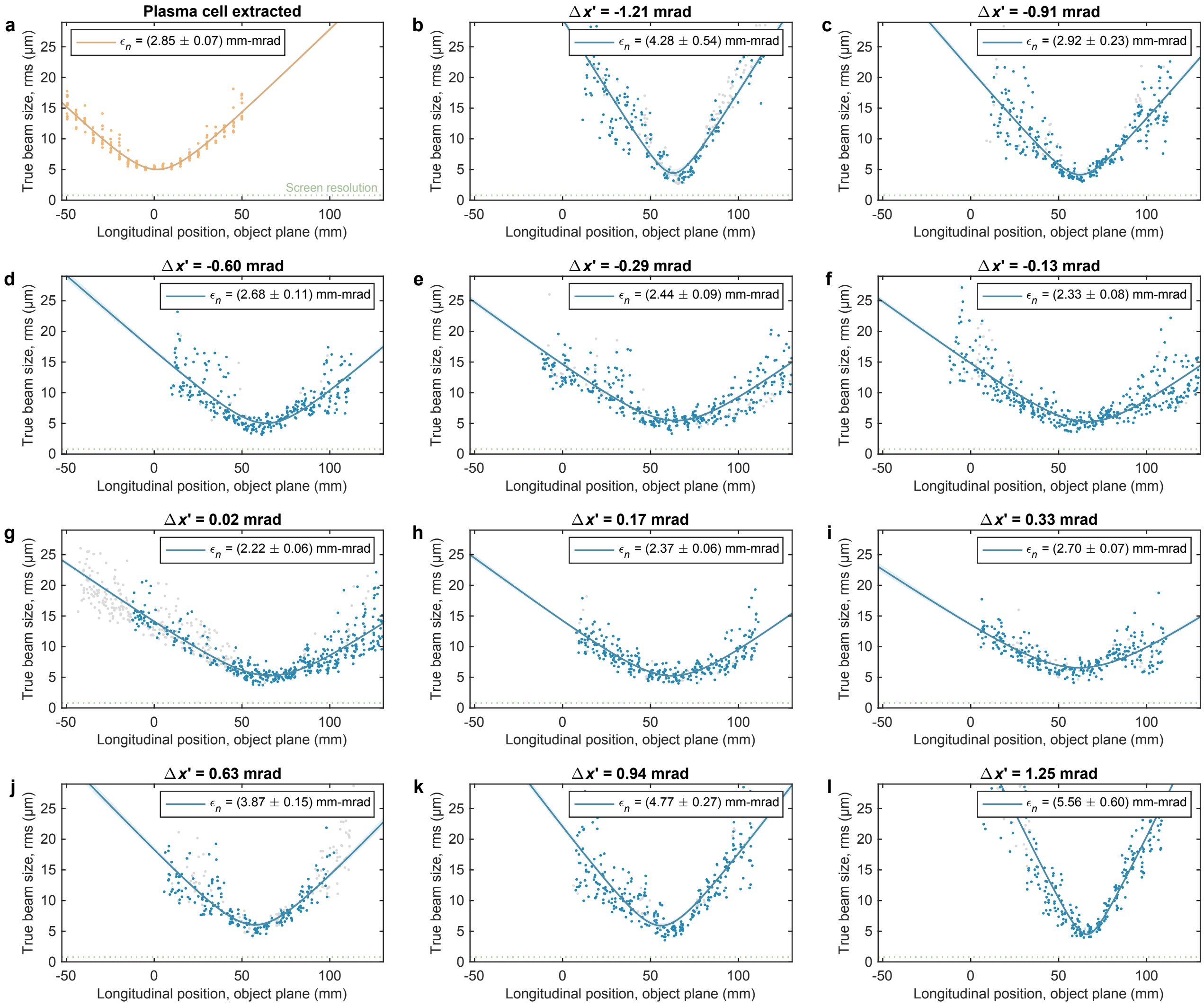

Supplementary Fig. 8 | Emittance measurements for various misalignments. a, An object-plane scan was performed while the plasma cell was extracted, measuring the beam size in the object plane for each shot (orange points). The quoted normalized emittance, ε_n, is found by a fit of the virtual waist (orange line). The screen resolution (green dotted line) is negligible. This is the same scan as shown in Fig. 2a. **b–l**, The measurement was repeated with the plasma cell inserted (blue points) for 11 separate values of $\Delta x'$, the angular misalignment between the driver and the trailing bunch. Shots are filtered (gray points) to ensure consistent input beams despite drift and jitter in bunch length (to be within ±15%) and charge (to be within ±5%), as well as a consistent scan range (to be within ±7.5 mm). These measurements correspond to the values in Fig. 3d.

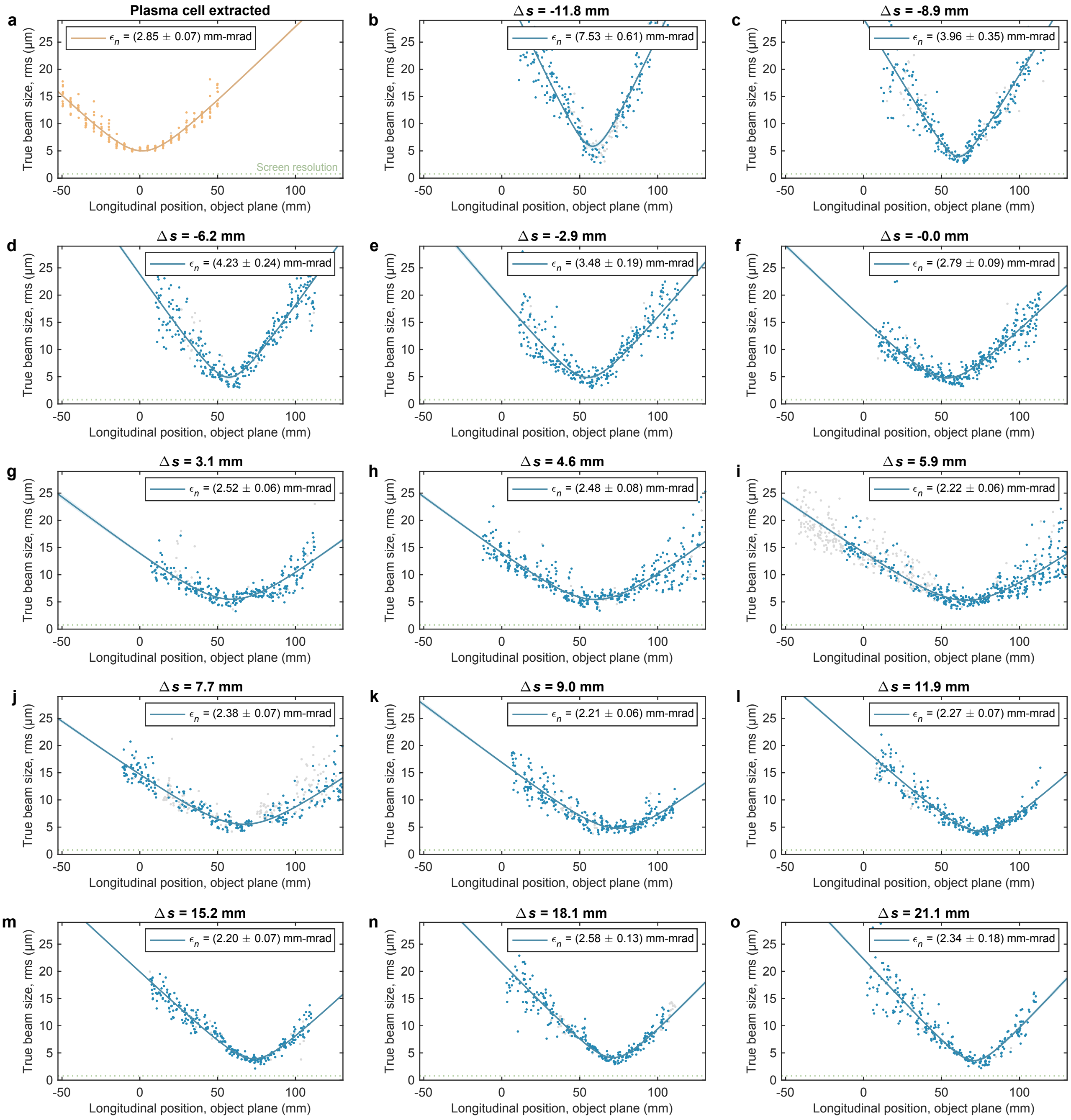

Supplementary Fig. 9 | Emittance measurements for various beam-waist locations. a, Similar to Supplementary Fig. 8, an object-plane scan was performed while the plasma cell was extracted, showing the measured beam size (orange points), the virtual-waist fit (orange line) and the negligible screen resolution (green dotted line). **b–o,** The beam size (blue points) was also measured with the plasma cell inserted for 14 different values of the trailing bunch's waist location Δ*s* relative to the plasma-cell entrance. Again, as in Supplementary Fig. 8, shots are filtered (gray points) to ensure consistency in bunch length (< ±15%), charge (< ±5%), and scan range (< ±7.5 mm). These measurements correspond to Fig. 4a.

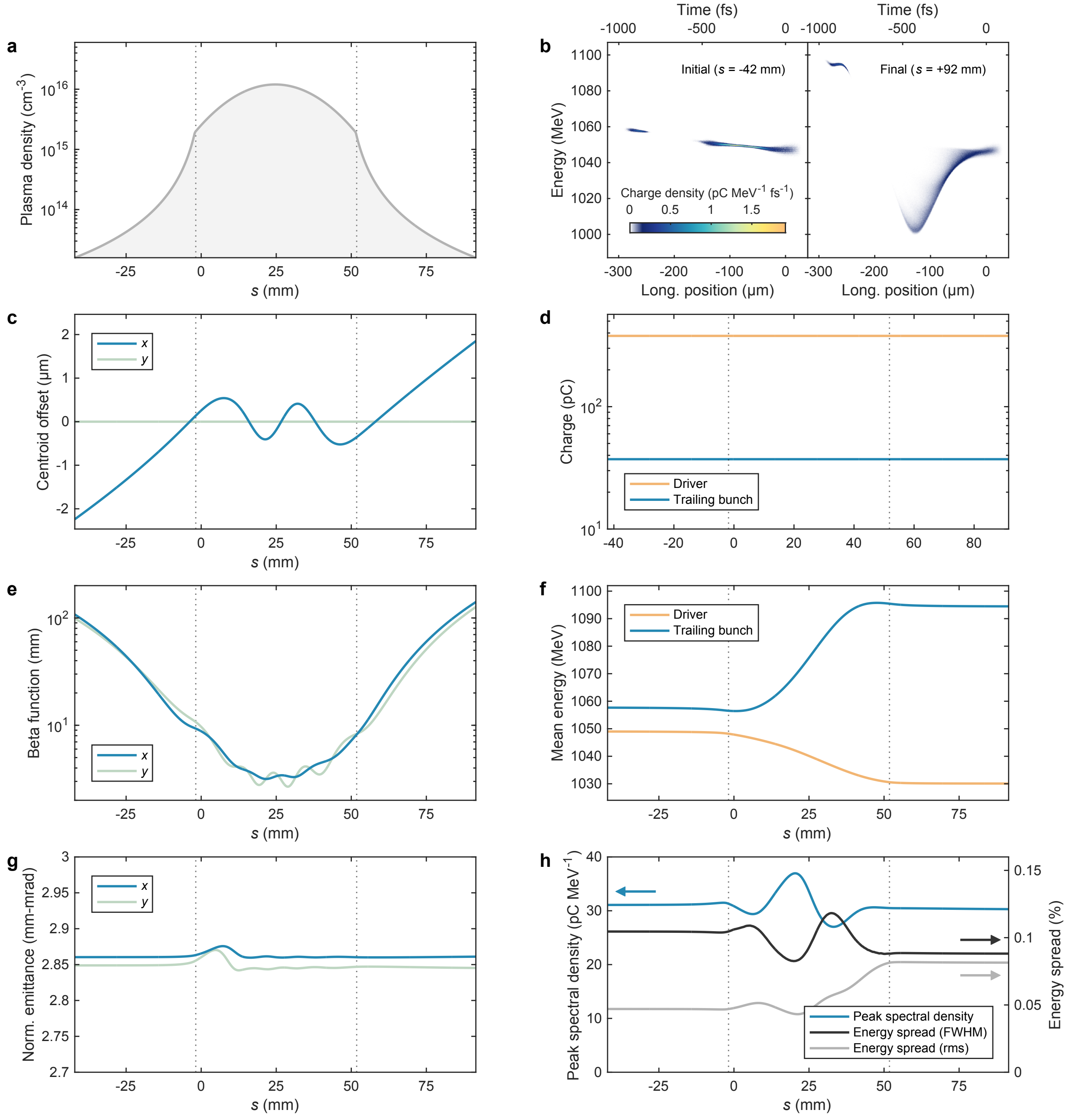

Supplementary Fig. 10 | Particle-in-cell simulation showing the evolution of the trailing-bunch properties. a, The longitudinal plasma-density profile, shown on a logarithmic scale, was estimated from measurements (Supplementary Fig. 4). Dotted vertical lines indicate the ends of the plasma cell, including its electrodes. **b**, The initial longitudinal phase space, based on the measurement (Supplementary Fig. 3), is shown in the left panel; the simulated final longitudinal phase space is shown in the right panel. **c**, The transverse centroid offset oscillates in the horizontal (*x*) plane, where an initial angular misalignment of 0.05 mrad is used (i.e., approximately the precision of the experiment). No misalignment is simulated in the vertical (*y*) plane. **d**, The charge of both the driver and trailing bunch is preserved throughout the simulation. **e**, Between the start and end of the simulation, the transverse phase space of the trailing bunch also evolved significantly, as indicated by the beta function, shown on a logarithmic scale. The beta functions in both the *x* and *y* planes were close to the matched beta function of the plasma accelerator: ~3 mm. **f**, Inside the plasma cell, the trailing-bunch particles gained 37 MeV of energy, whereas the driver lost 19 MeV, on average. This resulted in an energy-transfer efficiency of 19%, in agreement with measurements (Supplementary Fig. 6). **g**, As in the experiment, the normalized emittance of the trailing bunch was preserved in the horizontal plane (to within 0.1%). In the simulation, the emittance in the vertical plane was also preserved (also to within 0.1%); however, this quantity was not measured in the experiment. **h**, The energy spread of the trailing bunch, while changing during the acceleration process, was reduced finally from 0.10% to 0.09% FWHM, consistent with the experiment (Fig. 1f). While the peak spectral density does not decrease, the rms energy spread is seen to approximately double due to the generation of a low-energy tail (as seen in **b**).

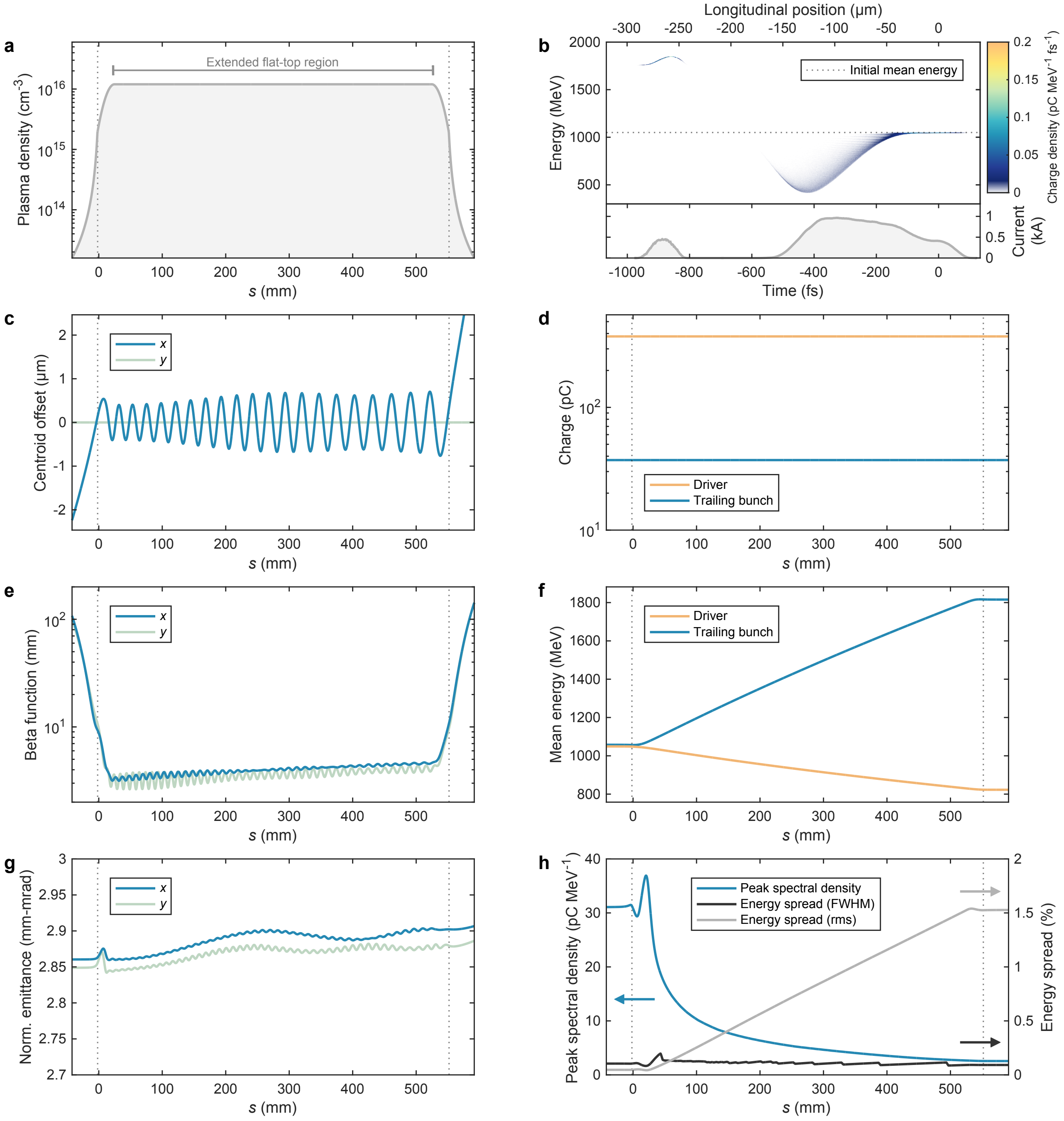

Supplementary Fig. 11 | Particle-in-cell simulation of an extended plasma cell. a, The longitudinal plasma-density profile is extended by adding a central 500-mm-long flat-top at 1.2×10^{16} cm^{-3}, while keeping the same up and down ramps. All initial beam parameters are identical to that of the simulation of the experiment shown in Supplementary Fig. 10. **b**, The resulting longitudinal phase space after acceleration is shown in the upper panel, in which the dotted line indicates the initial mean energy; the beam's current profile is shown in the lower panel. **c**, An initial 0.05-mrad angular misalignment causes the transverse centroid offset in the horizontal (x) plane to oscillate, with a normalized amplitude growth of 91%. **d**, The charge of both the driver and trailing bunch is preserved throughout the simulation. **e**, The beta function oscillates with a small amplitude around the matched value, which increases slowly with acceleration. **f**, The trailing-bunch particles gain a total of 758 MeV of energy, whereas the driver loses an average of 226 MeV per particle. In this extended cell, the energy-transfer efficiency is higher (at 33%) than in the shorter cell, as the beam is over-loaded in the flat-top: an effect that in the shorter cell was compensated by under-loading in the low-density ramps. **g**, Emittance is observed to be approximately preserved, increasing by only 1.6% in the horizontal plane and 1.3% in the vertical plane (both within the measurement error of 3%). Degradation due to hosing and beam-breakup instability is not observed. **h**, As a result of the over-loading of the field (seen in **b**), the rms energy spread increases significantly during acceleration, with a corresponding decrease in the peak spectral density. The FWHM energy spread also increases, but not as much (the jaggedness is caused by the finite resolution of the simulation). Arrows indicate the relevant vertical axis for each line.

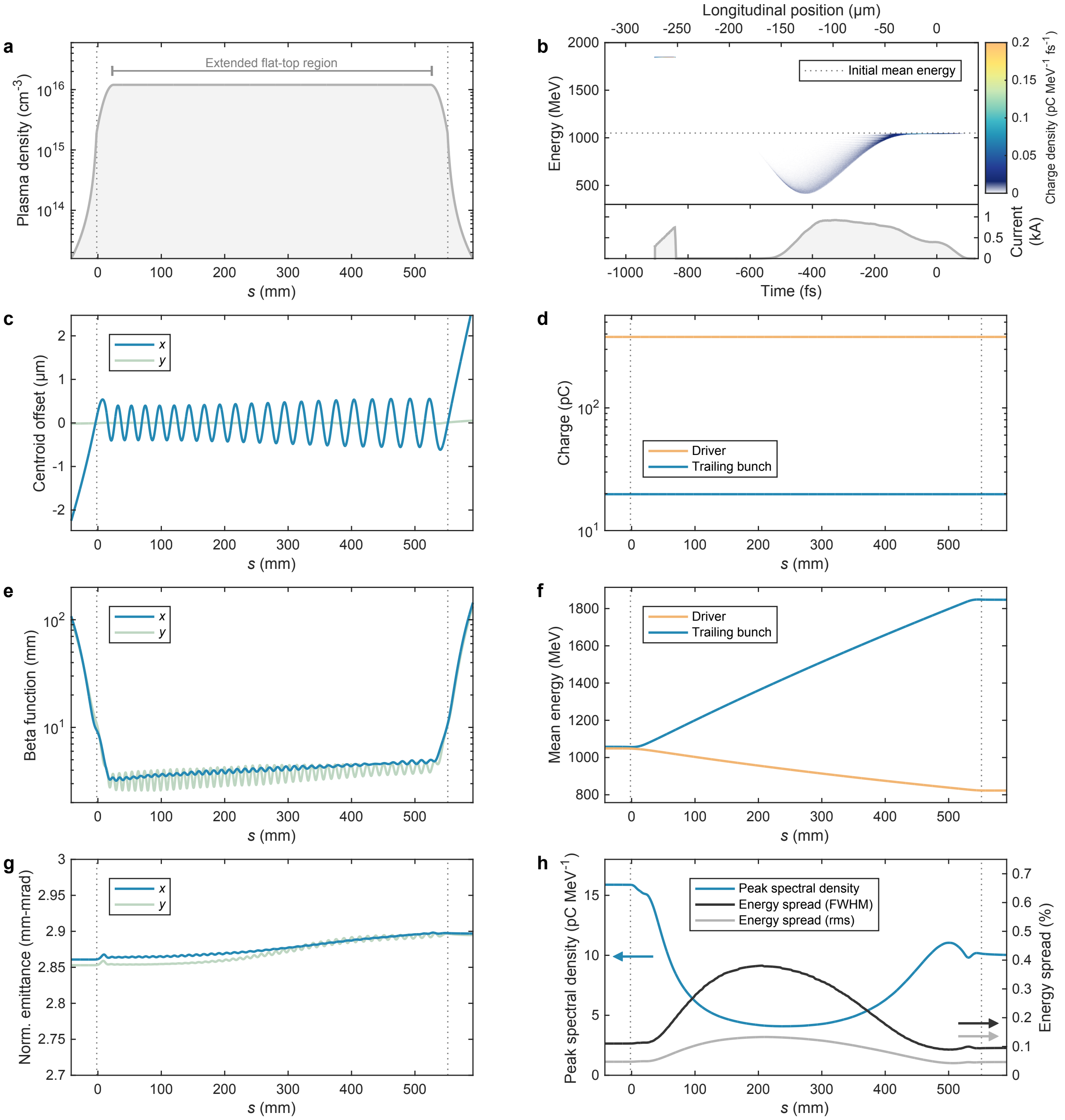

Supplementary Fig. 12 | Particle-in-cell simulation of optimal beam loading in an extended plasma cell. a, All plasma and driver parameters are identical to that of the extended simulation in Supplementary Fig. 11, except for the current profile of the trailing bunch. **b**, This optimized current profile is shown in the lower panel; the resulting final longitudinal phase space is shown in the upper panel, where the acceleration is seen to be uniform. **c**, A 0.05-mrad angular misalignment causes the transverse centroid offset in the horizontal (*x*) plane to oscillate, with a normalized amplitude growth of 47%. **d**, The charge of both the driver and trailing bunch is preserved throughout the simulation. **e**, The beta function remains approximately matched in *x*. **f**, The trailing bunch gains 790 MeV per particle, while the driver loses an average of 226 MeV per particle. In this simulation, the energy-transfer efficiency (at 18%) is approximately the same as in the experiment (Supplementary Fig. 10). **g**, Emittance is again approximately preserved, increasing by 1.3% and 1.5% in the horizontal and vertical planes, respectively (both within the 3% measurement error). **h**, Here, both the rms and FWHM energy spreads are preserved. Arrows indicate the relevant vertical axis for each line.

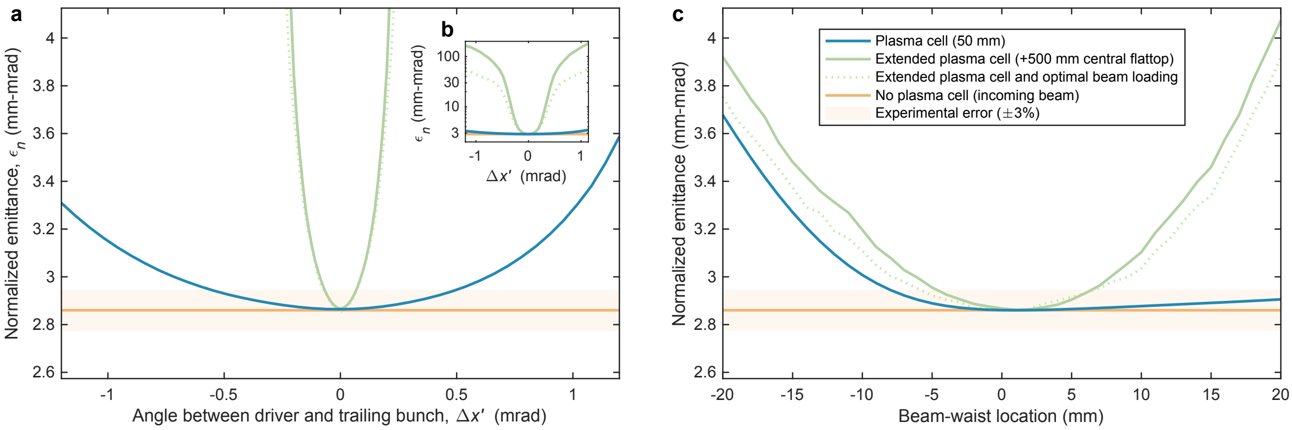

Supplementary Fig. 13 | Particle-in-cell simulations of the misalignment and mismatching scans. a, The outgoing normalized emittance is simulated as a function of angular misalignment between the driver and trailing bunch. The experimental results (see Fig. 3a) are qualitatively reproduced (blue line), showing emittance preservation with respect to the incoming beam (orange line) around zero misalignment. Extending the plasma-density profile with a 500 mm long central flattop but using the same beams gives a simulated outgoing emittance (solid green line) significantly higher at large misalignments, but is preserved to within the experimental measurement error (orange band) in a range ±0.07 mrad around zero—approximately corresponding to the alignment precision of the experiment. Simulations of optimally beam-loaded trailing bunches were also performed, matching the energy-transfer efficiency to the shorter plasma cell and preserving energy spread, showing a similar level of emittance growth—implying that transverse instabilities do not dominate. **b**, Inset showing a full, zoomed-out version of the same figure with a logarithmic vertical axis. **c**, The outgoing normalized emittance is also simulated as a function of beam-waist location. Again, the experimental results (see Fig. 4a) are qualitatively reproduced. The ±5 mm range of beam-waist locations for which emittance is preserved is within the precision achieved in the experiment.
